# Pregnancy programs epigenetic and transcriptional exhaustion in memory CD8^+^ T cells

**DOI:** 10.21203/rs.3.rs-2196637/v1

**Published:** 2023-04-05

**Authors:** Jared Pollard, Grace Hynes, Dengping Yin, Malay Mandal, Fotini Gounari, Maria-Luisa Alegre, Anita Chong

**Affiliations:** 1 Section of Transplantation, Department of Surgery, University of Chicago, Chicago IL, USA; 2 Section of Rheumatology, Department of Medicine, University of Chicago, Chicago IL, USA; 3 Department of Immunology, Mayo Clinic, Phoenix AZ, USA

## Abstract

Alloreactive memory T cells, unlike naive T cells, fail to be restrained by transplantation tolerance protocols or regulatory T cells, and therefore represent a critical barrier to long-term graft acceptance. Using female mice sensitized by rejection of fully-mismatched paternal skin allografts, we show that subsequent semi-allogeneic pregnancy successfully reprograms memory fetus/graft-specific CD8^+^ T cells (T_FGS_) towards hypofunction in a manner that is mechanistically distinct from naive T_FGS_. Post-partum memory T_FGS_ were durably hypofunctional, exhibiting enhanced susceptibility to transplantation tolerance induction. Furthermore, multi-omics studies revealed that pregnancy induced extensive phenotypic and transcriptional modifications in memory T_FGS_ reminiscent of T cell exhaustion. Strikingly, at loci transcriptionally modified in both naive and memory T_FGS_ during pregnancy, chromatin remodeling was observed exclusively in memory and not naive T_FGS_. These data reveal a novel link between T cell memory and hypofunction via exhaustion circuits and pregnancy-mediated epigenetic imprinting. This conceptual advance has immediate clinical relevance to pregnancy and transplantation tolerance.

## Introduction

Alloreactive memory T cells are key mediators of acute and chronic graft rejection and represent a potent barrier to transplantation tolerance in the clinic^1–4^. Alloreactive memory T cells can be generated by direct sensitization to foreign MHC (via prior transplantation or blood transfusion), or via heterologous immunity, whereby T cells primed during infections or exposure to environmental antigens are crossreactive to donor MHC^5–8^. While numerous therapies can successfully induce transplantation tolerance and cell-intrinsic hypofunction in naive T cells, memory T cells are resistant to these mechanisms^9–11^. Indeed, we recently reported that the presence of memory T cells sensitized to only a single donor antigen is sufficient to destabilize co-stimulation blockade-induced transplantation tolerance, underscoring the critical need to identify mechanisms for controlling immunological memory responses long-term^12^.

Mammalian pregnancy has long been recognized as a model of spontaneous alloantigen-specific tolerance, in which the maternal adaptive immune system must rapidly regulate responses towards the semi-allogeneic fetus to preserve fetal viability^13–15^. We previously reported that pregnancy can efficiently tolerize naive fetus-specific T cells, leading to the spontaneous acceptance of offspring-matched heart grafts in B cell-deficient post-partum mice^16^. This maternal T cell tolerance is characterized by the upregulation of coinhibitory markers, inhibition of pro-inflammatory cytokine production, and expansion of fetus-specific CD4^+^FoxP3^+^ T_regs_^16^. Lewis et al. recently demonstrated that naive OVA-reactive OT-I T cells acquire an exhausted transcriptional signature after pregnancy with OVA-expressing progeny^17^. However, the maternal immune response to the semi-allogeneic fetus in the presence of immunological memory, and more specifically whether semi-allogeneic pregnancy would be able to successfully restrain memory T cell responses, thus eliminating the barrier they pose to transplantation tolerance, remains largely unknown.

In this study, we define the tolerizing mechanisms of pregnancy on memory fetus/graft-specific CD8^+^ T cells (T_FGS_) that were generated by prior rejection of fully-mismatched paternal skin grafts. We report that sensitized female mice consistently achieve spontaneous tolerance towards the semiallogeneic fetus, resulting in pregnancy success rates comparable to those of naive mice. Remarkably, pregnancy reprogrammed memory T_FGS_ to a hypofunctional state that persisted post-partum, manifesting as enhanced susceptibility to co-stimulation blockade-mediated transplantation tolerance. Using high-dimensional multi-omics, we demonstrate that pregnancy induced distinct phenotypic and transcriptional profiles in naive versus memory T_FGS_, as well as an overlapping profile of exhaustion. Strikingly, we report divergent epigenetic fates of memory versus naive T_FGS_ during pregnancy: while the programming of T cell exhaustion was correlated with extensive chromatin remodeling in memory T_FGS_, these epigenetic modifications were absent in naive T_FGS_, even at loci of shared transcriptional modification. Collectively, this study highlights the evolutionary robustness of mammalian pregnancy in constraining not only naive but also memory fetus-specific CD8^+^ T cells, and introduces a novel mechanistic pathway that successfully reprograms memory CD8^+^ T cells towards hypofunction.

## Results

### Pregnancy induces a dysfunctional phenotype in memory T_FGS_

**Extended Data Fig. 1:**
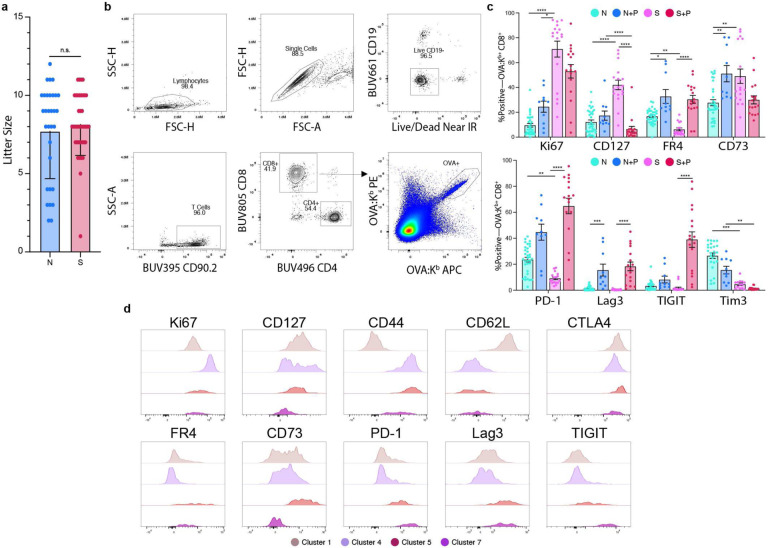
Memory and naive T_FGS_ are phenotypically modified by pregnancy. **a**, Bar graph showing litter sizes of Naive vs. Sensitized female mice achieving successful full-term pregnancies; *n* = 35–49 per group. **b**, Gating strategy for selection of OVA:K^b^ -specific CD8^+^ T cells (T_FGS_) via spectral flow cytometry. **c**, Bar graph showing showing percentages of of T_FGS_ cells from each experimental group expressing phenotypic activation and coinhibitory markers. Data acquired from 2 or more biologically independent experiments; *n* = 10–33 per group. **d**, Histograms showing phenotypic expression for FlowSOM clusters 1, 4, 5, and 7. Cluster 1 is predominantly Naive T_FGS_, cluster 4 is predominantly Sensitized T_FGS_, cluster 5 is shared by N+P and S+P T_FGS_, and cluster 7 is unique to S+P T_FGS_. Data represent mean ± SEM. P values were determined by Kruskal-Wallis test with Dunn’s post hoc test. ns: not significant; *P<0.05; **P<0.01; ***P<0.001; ****P<0.0001.

**Figure 1:**
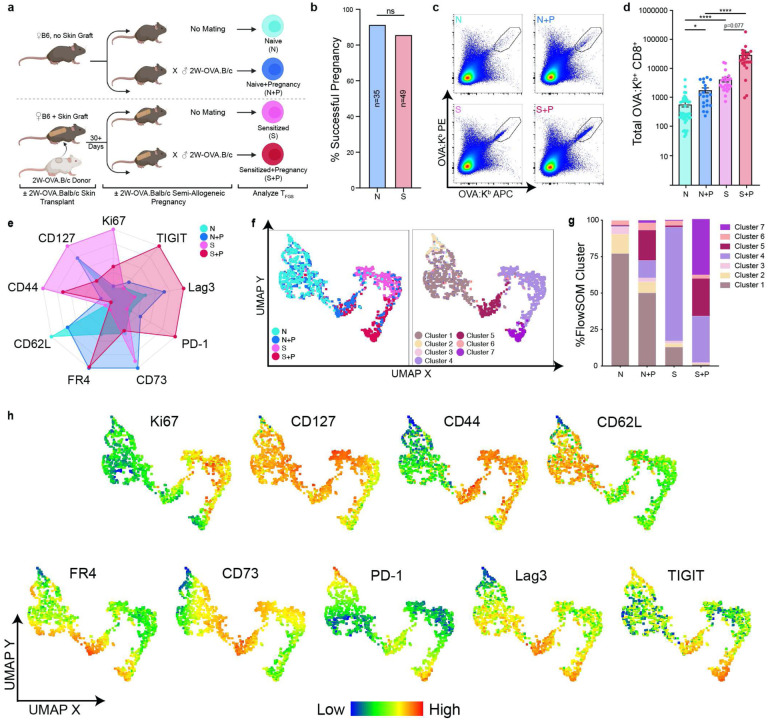
Post-partum memory T_FGS_ acquire a dysfunctional phenotype. **a**, Experimental design. Female B6 mice were mated with transgenic 2W-OVA.B/c mice, with or without sensitization to 2W-OVA.B/c via skin graft 30 days prior (S+P and N+P, respectively). Unmated mice with or without sensitization were included as controls (Sensitized and Naive, respectively). **b**, Bar graph showing percentage of Naive vs. Sensitized female mice achieving successful full-term pregnancies; *n* = 35–49 per group. Additionally, there was a 100% success rate in sensitized mice subjected to a b + second pregnancy (*n*=12) **c**, Representative pseudocolor plots showing OVA:K^b^ -specific CD8^+^ T cells (T_FGS_). **d**, Normalized total recovery of T_FGS_ cells. Data acquired from 2 or more biologically independent experiments; *n* = 20–38 per group. **e**, Radar plot showing phenotypic profile of T_FGS_ at markers of activation, memory, and coinhibition. Expression is represented as percentage of the highest-expressing group for each marker. **f**, UMAP with experimental groups (left) and FlowSOM clustering (right) reveals distinct phenotypic subsets in T_FGS_. **g**, Stacked bar graph showing FlowSOM cluster distributions for each experimental group. **h**, UMAP with heatmap overlays to show expression of each phenotypic marker on T_FGS_ at single-cell resolution. Data represent mean ± SEM. P values were determined by Kruskal-Wallis test with Dunn’s post hoc test. ns: not significant; *P<0.05; ****P<0.0001. Statistical analysis of this flow dataset can be found in Extended Data Fig. 1.

To test whether semi-allogeneic pregnancy is possible in females harboring immunological memory to paternal antigens, we sensitized female C57Bl/6 (B6, H-2^b^) mice with male 2W-OVA.BALB/c (H-2^d^) skin transplantation (skinTx). Donor 2W-OVA.BALB/c mice constitutively express 2W-OVA, a recombinant model antigen, allowing endogenous OVA-specific CD8^+^ T cells from the recipient to be tracked via flow cytometry using fluorophore-labeled OVA:K^b^ tetramers^18,19^. Recipient B6 mice rejected skin grafts within 10 days (data not shown), and at day 30+ post-transplantation, skin-sensitized females were mated with 2W-OVA.BALB/c males (Sensitized+Pregnancy) (Fig. 1a). The rates of successful pregnancy, including multiple successive pregnancies, were comparable between Sensitized+Pregnancy (S+P) and Naive+Pregnancy (N+P) mice, and there were no differences in the resulting litter sizes (Fig. 1b, Extended Data Fig. 1a). Thus, we concluded that pregnancy successfully modified memory T_FGS_ to prevent rejection of the semi-allogeneic fetus.

To investigate the effects of pregnancy on naive vs. memory T_FGS_, we expanded our experimental model by including Naive and Sensitized mice without mating or pregnancy (Fig. 1a). We designed a 19 color spectral flow cytometry panel to profile T_FGS_ phenotypes across multiple activation, memory, and inhibitory markers (Supplementary Table 1). Using this panel, we analyzed OVA-specific CD8^+^ T cells at day 30+ post-skinTx for Sensitized mice, or at post-partum day 0–3 for N+P and S+P mice. We observed a significant increase in T_FGS_ recovery from S+P mice compared to N+P mice (Fig. 1–d). Despite this expansion, S+P T_FGS_ displayed elevated expression of multiple coinhibitory markers compared to Sensitized T_FGS_, including PD-1, LAG3, TIGIT, and FR4 (Fig. 1e, Extended Data Fig. 1–c). N+P T_FGS_ also upregulated FR4, CD73, and LAG3 compared to Naive T_FGS_.

To visualize T_FGS_ phenotypes at single-cell resolution, we conducted uniform manifold approximation and projection (UMAP) dimensionality reduction along with FlowSOM clustering, which resulted in the identification of 4 major and 3 minor clusters (Fig. 1f). As anticipated, Naive and Sensitized T_FGS_ were largely homogenous, with >75% of these cells mapping to Cluster 1 or Cluster 4, respectively (Fig. 1g). In contrast, the effect of pregnancy on T_FGS_ was heterogeneous, with ~50% of N+P and ~25% of S+P T_FGS_ remaining phenotypically similar to Naive or Sensitized T_FGS_, respectively (Fig. 1g). Notably, Cluster 5 was identified as a shared cluster of pregnancy, comprising ~25% of both N+P and S+P T_FGS_ and defined by elevated expression of multiple coinhibitory markers, along with reduced expression of the proliferation marker Ki67 (Fig. 1–h, Extended Data Fig. 1–d). Cluster 7 was unique to S+P T_FGS_; this cluster was similar to Cluster 5 with the exception of reduced CD73 expression (Fig. 1h, Extended Data Fig. 1–d). Collectively, the phenotypic similarities observed between the Cluster 5 and Cluster 7 raise the possibility that pregnancy programs hypofunction into naive and memory T_FGS_ through the induction of a shared set of modifications associated with anergy and exhaustion.

### Pregnancy elicits a shared transcriptional signature in memory and naive T_FGS_

**Extended Data Fig. 2:**
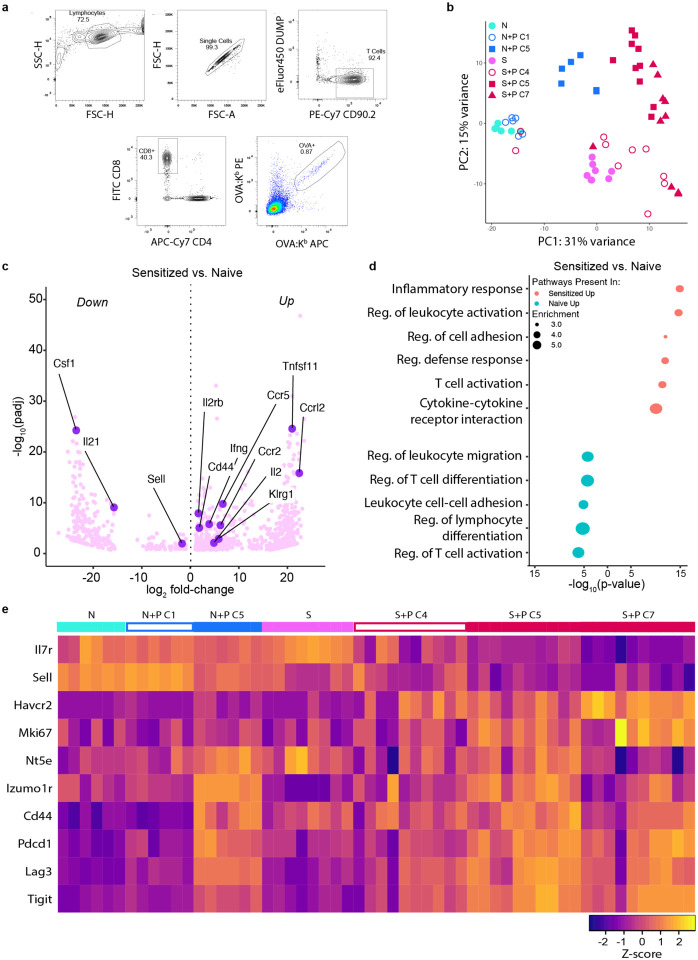
Gating strategy and transcriptional analysis of T_FGS_ subsets. **a**, Gating strategy for selection and fluorescent cell sorting of OVA:K^b^-specific CD8^+^ T cells (T_FGS_) via flow cytometry. **b,** PCA plot comparing T_FGS_ transcriptional profiles corroborates the UMAP shown in Fig. 2b and captures a high percentage of variance in PC1 and PC2. **c-d,** Volcano plot (**c**) and Metascape pathway analysis (**d**) of DEGs for Sensitized vs. Naive T_FGS_. **e**, Row-normalized RNA-seq expression of the loci analyzed by flow cytometry in Fig. 1.

**Figure 2:**
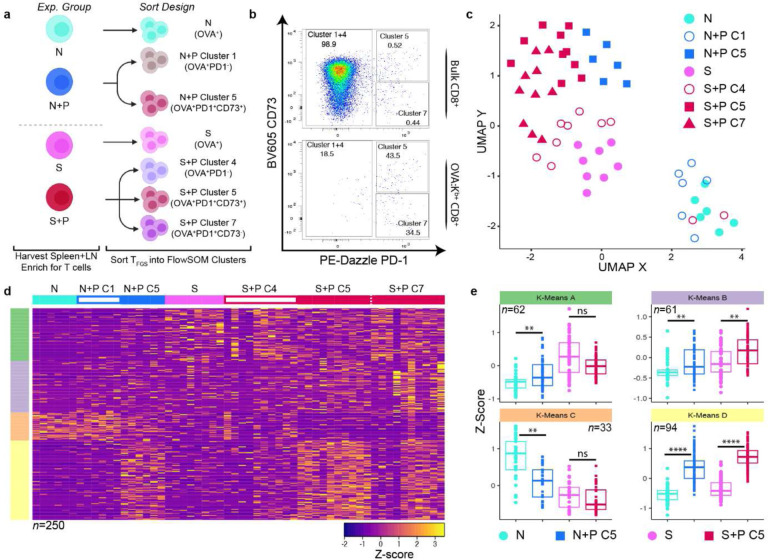
Pregnancy induces broad transcriptional modifications in memory and naive T_FGS_. **a**, Sorting strategy. T_FGS_ subsets for each experimental group were flow-sorted into their most prevalent phenotypic subsets as observed via FlowSOM in Fig. 1F. T_FGS_ were acquired and sorted from 2 biologically independent experiments; *n* = 3–5 per group, with technical replicates for each biological sample. **b**, Representative pseudocolor plots showing the distributions of Cluster 1+4, Cluster 5, and Cluster 7 within b + bulk or OVA:K -specific Sens+Preg CD8 T cells. The percentage of each cluster within T_FGS_ is comparable to the distribution of our FlowSOM analysis in Fig. 1g for each experimental group. **c**, UMAP plot comparing transcriptional profiles between sorted T_FGS_ subsets. **d**, Row-normalized RNA-seq expression of the top differentially expressed genes (*n*=250) among T_FGS_ subsets. Expression was organized into four subsets via K-means clustering, indicated by left-side color column (K-Means A-D). **e**, Box plots visualizing relative expression of DEGs in each K-Means cluster identified in (**d**). Minimum criteria for all DEGs shown in this figure was both q<0.1 and log_2_ fold-change>0.9. P values were determined by Kruskal-Wallis test with Dunn’s post hoc test (**d**). ns: not significant; **P<0.01; ****P<0.0001.

We next tested the hypothesis that the shared set of phenotypic markers induced by pregnancy in both naive and memory T_FGS_ was indicative of a broader set of shared transcriptional modifications. Therefore, we performed genome-wide transcriptional profiling of T_FGS_ by designing a cell sorting panel that allowed us to sort T_FGS_ into the 4 predominant phenotypic subsets observed in post-partum T_FGS_: Cluster 1 (C1, naive-like phenotype), Cluster 4 (C4, sensitized-like phenotype), Cluster 5 (C5, shared by N+P and S+P), and Cluster 7 (C7, unique to S+P) (Fig. 2a). The proportions of each cluster in this panel were consistent with our original phenotypic data (Extended Data Fig. 2b, Extended Data Fig. 2a). This approach allowed us to address the heterogeneity among pregnancy-modified T_FGS_ while also retaining the sequencing depth of bulk RNA-sequencing.

**Extended Data Fig. 3:**
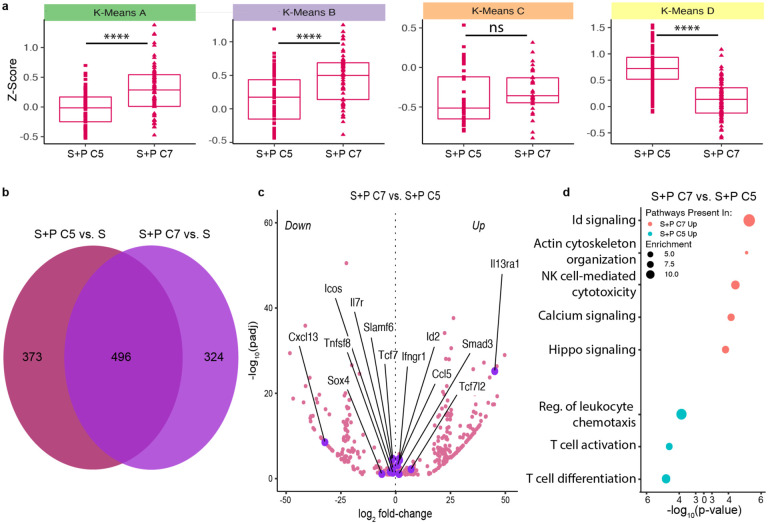
Comparison of S+P C7 vs. S+P C5 T_FGS_ subsets. **a**, Box plots visualizing S+P C7 vs. S+P C5 expression of DEGs in each K-Means cluster identified in Fig. 2D. **b-d,** Venn diagram (**b**), volcano plot (**c**) and Metascape pathway analysis (**d**) of DEGs for S+P C7 vs. S+P C5 T_FGS_ subsets. Minimum criteria for all DEGs shown in this figure was both q<0.1 and log_2_ fold-change>0.9. P values were determined by two-tailed Mann-Whitney test (**d**). ns: not significant; ****P<0.0001.

With this RNA-seq dataset, we first constructed a heatmap to visualize the transcriptional expression of the markers used in our flow cytometry panel in Figure 1 and found that the expression patterns in our transcriptional dataset were consistent with phenotypic data (Extended Data Fig. 2e). We then performed differential expression analysis across 17,659 genes and visualized the global transcriptional differences via UMAP and principal component analysis (PCA) dimensionality reduction (Fig. 2c and Extended Data Fig. 2b). As expected, Sensitized and Naive T_FGS_ displayed distinct transcriptional signatures, with many genes and pathways for T cell activation and effector function upregulated in Sensitized T_FGS_ (Fig. 2c, Extended Data Fig. 2–d). Furthermore, the N+P C1 subset was nearly identical to Naive T_FGS_, and the S+P C4 subset was similar to Sensitized T_FGS_ (Fig. 2c), corroborating our above conclusion that a subset of T_FGS_ remains unmodified by pregnancy in both Naive and Sensitized mice. Remarkably, the global transcriptional profile between S+P C5 and N+P C5 appeared to be similar via UMAP and heatmap, indicative of considerable transcriptional overlap between post-partum naive and memory T_FGS_ (Fig. 2–e). We next analyzed the differentially expressed genes (DEGs) between S+P C7 vs. C5 T_FGS_ and observed that these populations were more similar at the genome-wide level than initially anticipated based on our phenotypic data (Extended Data Fig. 3b). However, modest transcriptional differences were indeed present between these subsets, including enrichment of Inhibitor of DNA binding (Id) signaling and NK cell activation pathways in S+P C7 (Extended Data Fig. 3–d).

**Figure 3:**
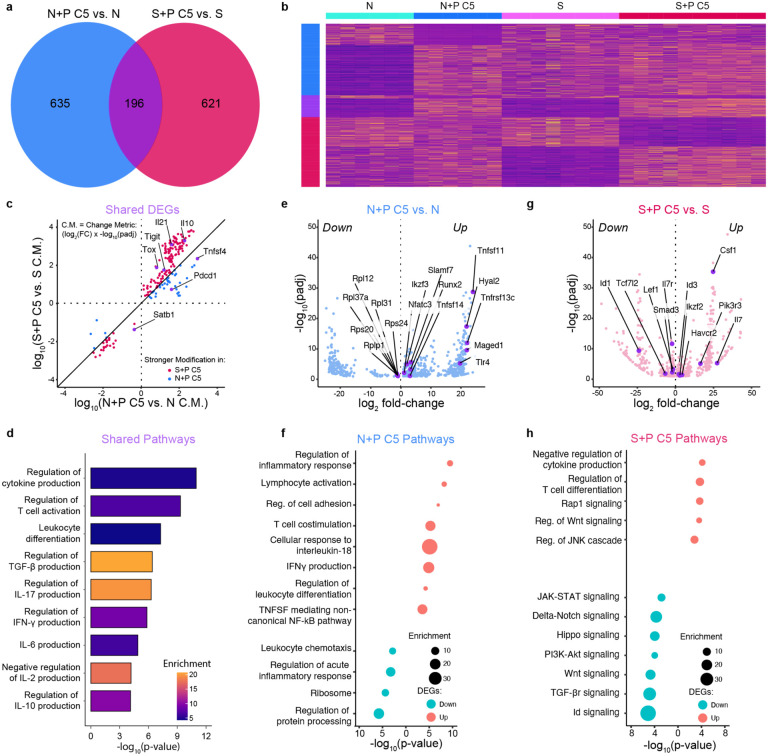
Post-partum memory and naive T_FGS_ are distinct, but share a core transcriptional signature. **a**, Venn diagram of the shared vs. unique transcriptional modifications induced in T_FGS_ by pregnancy. **b**, Row-normalized RNA-seq expression of the differentially expressed genes that are unique or shared by N+P C5 vs. N, or S+P C5 vs. S T_FGS_ subsets. **c**, Dot plot comparing relative expression of shared DEG’s between N+P C5 and S+P C5. The Change Metric (C.M.) is a single statistic that merges FDR-corrected p-value and log fold change (±log_2_(FC) × -log(FDR)). **d**, Metascape pathway analysis for shared DEG’s between N+P C5 and S+P C5 (**a**). **e-f**, Volcano plot (**e**) and Metascape pathway analysis (**f**) for DEG’s unique to N+P C5 vs. Naive T_FGS_. **gh**, Volcano plot (**g**) and Metascape pathway analysis (**h**) for DEG’s unique to S+P C5 vs. S T_FGS_.

To directly compare the effect of pregnancy on naive vs. memory T_FGS_, we examined the set of DEGs induced between N+P C5 vs. Naive T_FGS_, and of S+P C5 vs. Sensitized T_FGS_ (Fig. 3–b). This analysis revealed a core signature of transcriptional modification that was shared between naive and memory T_FGS_ after pregnancy (n=196 shared DEGs), the majority of which were upregulated (168 genes) rather than downregulated (28 genes) (Fig. 3c). Notable examples of shared upregulated genes included *Tox*, *Lag3*, *Tigit*, *Nfatc1*, *Nfat5*, *Tnfsf4*, *Ctla4*, and *Pdcd1*, whereas *Satb1* was downregulated by pregnancy. Additionally, Metascape pathway analysis of these shared DEGs revealed an enrichment for multiple T cell immunomodulatory pathways, including IL-6, IL-10, TGF-β, and IFN-γ (Fig. 3–d). Although the relative magnitude of transcriptional change was greater in S+P C5 than N+P C5 T_FGS_ for the majority of shared DEGs, notable exceptions included *Pdcd1* (PD-1) and *Tnfsf4* (OX40L), which were more upregulated in N+P C5 (Fig. 3c).

**Extended Data Fig. 4:**
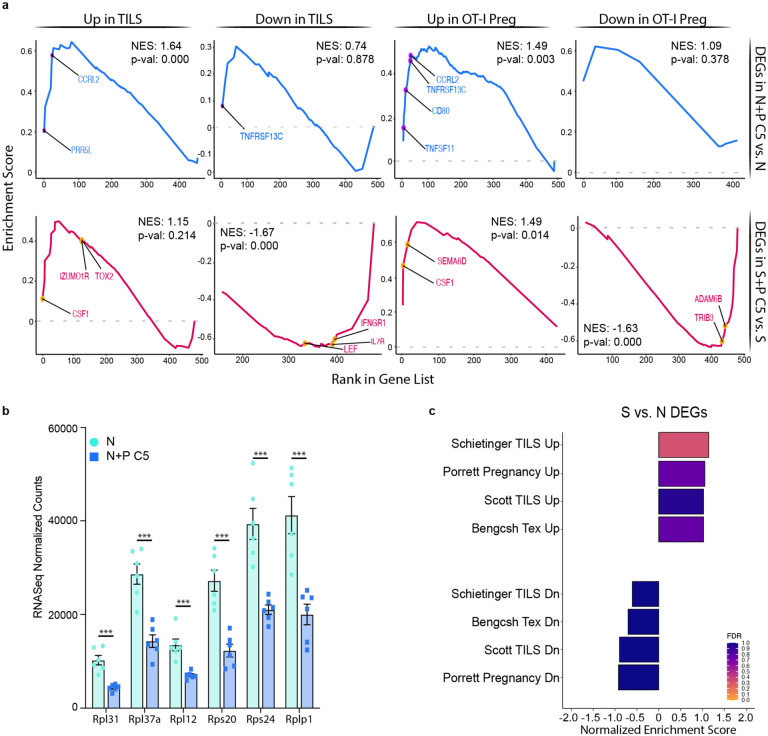
Similarities and differences between post-partum naive vs. memory T_FGS_. **a,** GSEA curves showing enrichment of dysfunctional T cell signatures during tumor responses (TILS) and pregnancy (Scott et al. 2019; Lewis et al. 2022) in N+P C5- and S+P C5-induced transcriptional modifications. NES, Normalized Enrichment Score. **b**, Bar graph of normalized RNA-Seq read counts for N+P C5 vs. Naive DEGs in the Ribosome Pathway (Kegg mmu03010). Differential expression of these genes was not observed in S+P C5 vs. Sensitized T_FGS_. **c**, Summary of GSEA analysis comparing Sensitized vs. Naive DEGs to published gene sets of exhaustion. P values were determined by Wald’s test. *P<0.05; ***P<0.001; ****P<0.0001.

Despite the substantial transcriptional overlap, the majority of pregnancy-induced DEGs were actually unique to either N+P C5 or S+P C5 T_FGS_ (Fig. 3a). Pathway analysis of these DEGs indicated that many of the uniquely expressed genes also belonged to pathways of T cell activation and cytokine production (Fig. 3–h). Notably, Lewis et al. recently reported that pregnancy-induced hypofunction in naive OT-I cells was characterized in part by translational repression, observed via downregulation of ribosom-eassociated genes^17^. We corroborate this finding in endogenous N+P C5 T_FGS_, whereas enrichment of this pathway was not observed in S+P C5 T_FGS_ (Fig. 3–h, Extended Data Fig. 4b). These data suggest that while a core transcriptional signature is consistently induced by pregnancy in T_FGS_, the majority of transcriptional modifications induced by pregnancy were unique to naive or memory T_FGS_, and these may additionally contribute to distinct mechanisms of enforcing T cell hypofunction in memory vs. naive T_FGS_.

### Post-partum memory T_FGS_ acquire an exhausted transcriptome and phenotype

**Figure 4:**
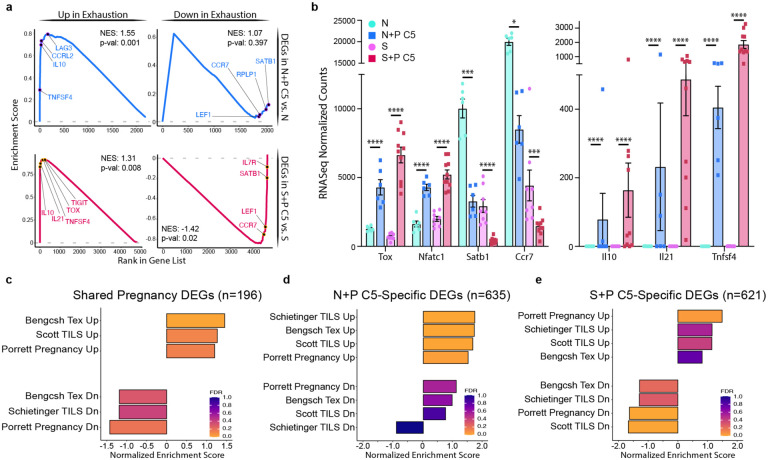
Post-partum memory and naive T_FGS_ acquire transcriptional signatures of exhaustion. **a**, GSEA curves showing enrichment of the exhausted T cell signature (chronic viral infection; Bengsch et al. 2018) in N+P C5- and S+P C5-induced transcriptional modifications. NES, Normalized Enrichment Score. **b**, Bar graphs of normalized RNA-Seq read counts for selected pregnancy-induced DEGs. **c-e**, Summary of GSEA analysis comparing shared DEGs **(c)**, unique N+P C5 vs. Naive DEGs (**d**), or unique S+P C5 vs. Sensitized DEGs (**e**) to published gene sets of exhaustion. P values were determined by Wald’s test. *P<0.05; ***P<0.001; ****P<0.0001.

The observation that pregnancy programs an exhaustion-associated transcriptional signature in naive OT-I CD8^+^ T cells^17^, along with our observation of a shared transcriptional core between post-partum memory and naive T_FGS_, led us to consider the hypothesis that pregnancy reprograms memory T_FGS_ towards a transcriptional state of exhaustion. To this end, we ranked the DEGs in N+P C5 vs. Naive and S+P C5 vs. Sensitized T_FGS_, comparing them to hallmark gene sets of exhausted CD8^+^ T cells (T_ex_) during chronic infection via Gene Set Enrichment Analysis (GSEA)^22–24^. Indeed, we observed a significant enrichment in the T_ex_ signature in both S+P C5 vs. Sensitized and N+P C5 vs. Naive DEGs (Fig. 4–b). Notably, both *Lag3* and *Tigit* were identified as part of the leading edge in our Gene Set Enrichment Analysis (Fig. 4b). This finding was corroborated with multiple additional T_ex_ gene sets from cancer, chronic infection, and pregnancy (Fig. 4–e). As a control, we ran GSEA on the DEGs of Sensitized vs. Naive T_FGS_; as expected, there was no enrichment for any exhaustion gene set in these DEGs (Extended Data Fig. 4c).

We also conducted separate GSEA analyses on the unique and shared DEGs between N+P C5 and S+P C5, and observed an enrichment of the upregulated T_ex_ signature within the set of 196 DEGs shared by N+P C5 and S+P C5 (Fig. 4c). Interestingly, the T_ex_ signature was significantly enriched for only the upregulated genes unique to N+P C5, whereas both upregulated and downregulated genes unique to S+P C5 were enriched for the T_ex_ signature (Fig. 4–e, Extended Data Fig. 4a). Taken together, these data confirm that pregnancy induces a partially overlapping signature of transcriptional T cell exhaustion in both naive and memory T_FGS_, but also provide new evidence suggesting that pregnancy utilizes additional distinct mechanisms to program naive vs. memory T_FGS_.

**Extended Data Fig. 5:**
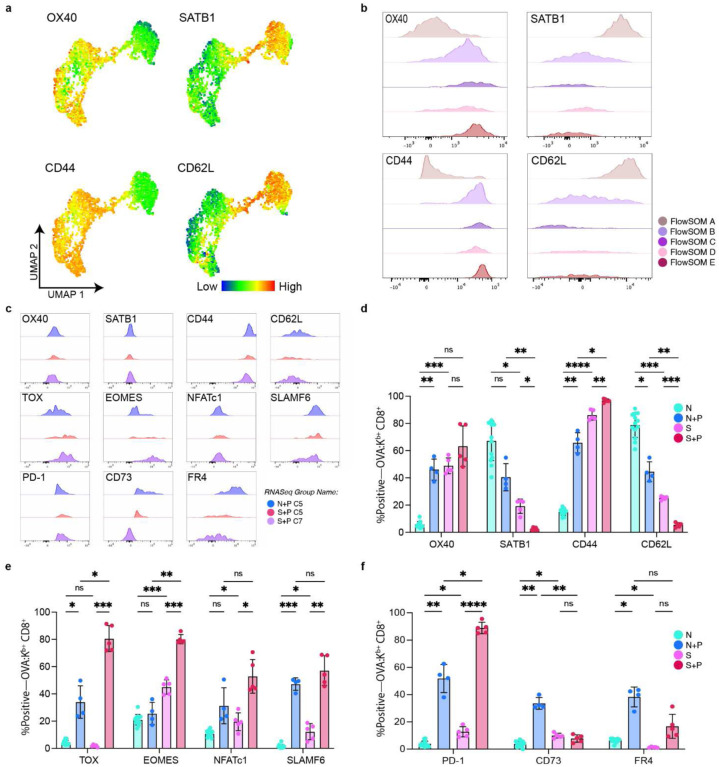
Updated phenotypic panel enhances separation of post-partum memory vs. naive T_FGS_. **a**, UMAP with heatmap overlays of additional phenotypic markers expressed by TFGS across experimental groups. **b**, Additional histograms showing phenotypic expression for FlowSOM clusters from Fig. 4b. FlowSOM A is predominantly Naive T_FGS_, FlowSOM B is predominantly Sensitized T_FGS_, FlowSOM C+D are predominantly S+P T_FGS_, and FlowSOM E is unique to N+P T_FGS_. **c**, Histograms showing phenotypic expression of new markers within our original FlowSOM Clusters that were used for RNA-Seq sorting. **d-f,** Bar graphs showing percentages of T_FGS_ cells expressing phenotypic markers of activation and exhaustion. Data acquired from 2 or more biologically independent experiments; *n* = 4–13 per group. Data represent mean ± SEM. P values were determined by Kruskal-Wallis test with Dunn’s post hoc test. ns: not significant; *P<0.05; **P<0.01; ***P<0.001; ****P<0.0001.

**Figure 5:**
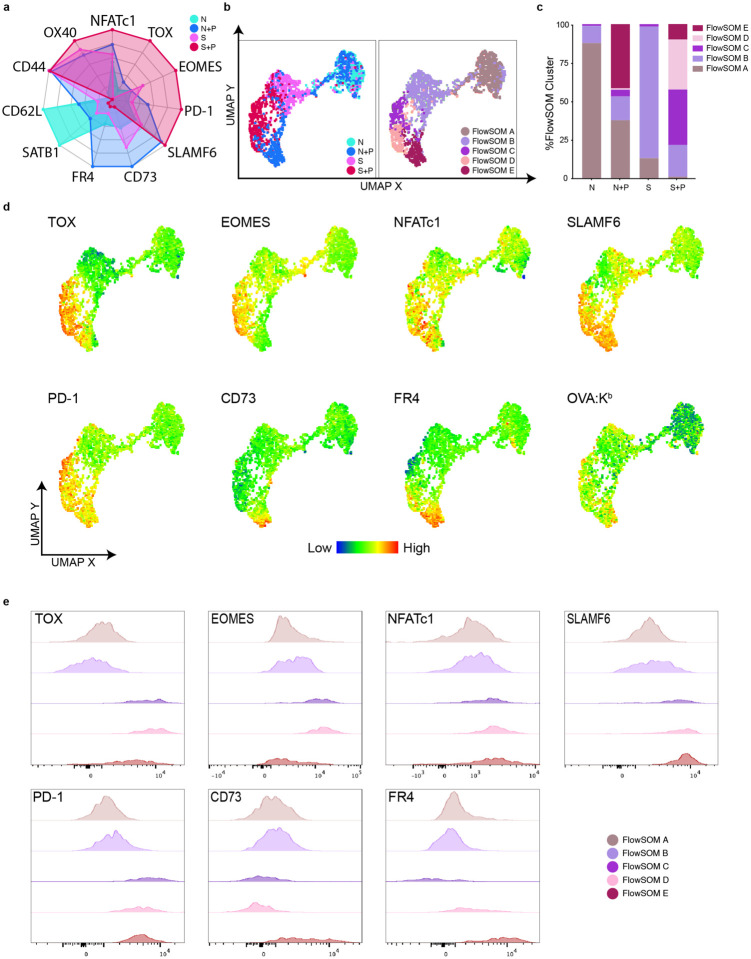
Pregnancy programs distinct and overlapping exhaustion/dysfunction profiles in memory and naive T _FGS_. **a-e**, New flow cytometry panel based on RNA-seq results confirms phenotypic exhaustion in post-partum T _FGS_. **a,** Radar plot presenting relative expression of phenotypic markers demonstrates enhanced separation between N+P and S+P T_FGS_. **b-c,** UMAP and FlowSOM reveal distinct clusters for N+P and S+P T_FGS_ driven by phenotypic differences in TOX, EOMES, FR4, and CD73. **d,** UMAP with heatmap overlays were generated to visualize phenotypic differences between T_FGS_ subsets. **e**, Histograms showing phenotypic differences between FlowSOM clusters. Flow cytometry data were acquired from 2 or more biologically independent experiments; *n* = 4–13 per group. Statistical Analysis of this flow dataset can be found in Extended Data Fig. 5.

We next sought to validate the transcriptional findings with phenotypic evidence. We designed a larger 23-color spectral flow cytometry panel to assess the phenotypic expression of additional markers identified in our transcriptional analysis (Supplementary Table 1). First, we confirmed the presence of an overlapping phenotypic signature between N+P and S+P T_FGS_, characterized by the upregulation of NFATc1, SLAMF6, OX40, and PD-1, along with downregulation of SATB1 (Fig. 5–d, Extended Data Fig. 5). Importantly, this new panel more readily captured differences between N+P and S+P T_FGS_, illustrated by radar plot and by UMAP+FlowSOM (Fig. 5–d, Extended Data Fig. 5–b). N+P T_FGS_ predominantly mapped to Cluster E, and preferentially upregulated FR4 and CD73 compared to S+P T_FGS_ (Fig. 4d–e, Extended Data Fig. 5–f). Conversely, Cluster E was a minor subset of S+P T_FGS_, and the majority of S+P T_FGS_ mapped to Clusters C+D, which were characterized by a significantly more robust induction of TOX and EOMES compared to N+P T_FGS_ (Fig. 5–e, Extended Data Fig. 5–f). Collectively, these data illustrate the gradation of shared and distinct markers of phenotypic and transcriptional exhaustion induced by pregnancy in naive and memory CD8^+^ T_FGS_.

### Pregnancy induces distinct states of hypofunction in naive vs. memory T_FGS_

The broad phenotypic and transcriptional modifications consistent with T cell exhaustion prompted us to test if pregnancy induced cell-intrinsic hypofunction in memory T cells^16,17,20,21^. We first quantified the in-vitro cytokine production capability of CD8^+^ T cells following stimulation with allogeneic APCs. As expected, ~12% and 30% of Sensitized T_FGS_ produced TNF-α and IFN-γ, respectively (Fig. 6a). In contrast, we found that N+P T_FGS_ exhibited minimal TNF-α and IFN-γ production, remaining comparable to Naive T_FGS_ (Fig. 6a). Furthermore, pregnancy significantly but incompletely reduced TNF-α production in S+P T_FGS_ compared to Sensitized T_FGS_, but did not significantly reduce IFN-γ production (Fig. 6a). To test for cell-intrinsic hypofunction of memory T_FGS_ *in vivo*, we adoptively transferred (AdTr) purified CD8^+^ T cells from Sensitized or S+P mice into naive B6 hosts, followed by transplantation with a semi-allogeneic F1 heart graft (B6×2W-OVA.BALB/c) along with a tolerance-inducing regimen of donor splenocyte transfusion and anti-CD154 costimulation blockade (CoB) (Fig. 6c). Consistent with previous reports^9,11^, memory CD8^+^ T cells from Sensitized mice prevented stable tolerance induction (Fig. 6d). Remarkably, CoB induced a significant extension of allograft survival in recipients of S+P CD8^+^ T cells compared to those receiving Sensitized CD8^+^ T cells, with ~50% of the grafts surviving long-term (Fig. 6d); Thus, we conclude that pregnancy is capable of enforcing a cell-intrinsic state of hypofunction not only in naive T_FGS_, but also in memory T_FGS_.

**Extended Data Fig. 6:**
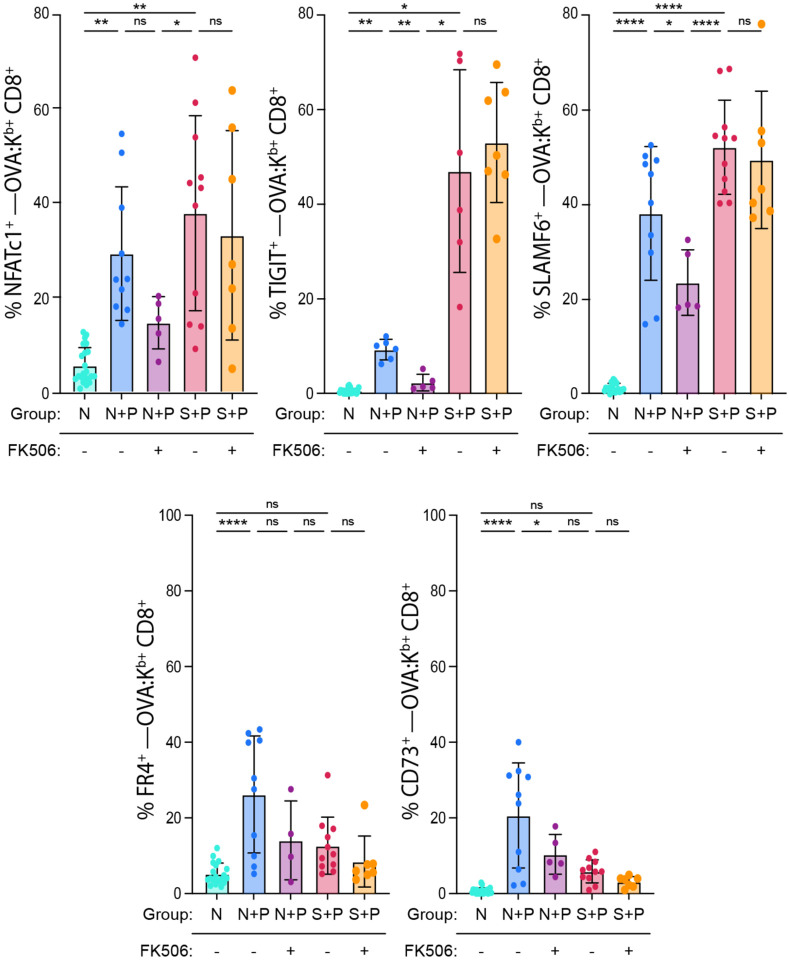
S+P T_FGS_ resist FK506-induced phenotypic modifications. Bar graphs showing percentages of T_FGS_ cells expressing phenotypic markers of activation and exhaustion in the presence or absence of FK506, an inhibitor of NFAT. Data acquired from 2 or more biologically independent experiments; *n* = 4–13 per group. Data represent mean ± SEM. P values were determined by ordinary one-way ANOVA or Brown-Forsythe and Welch ANOVA. ns: not significant; *P<0.05; **P<0.01; ****P<0.0001.

The distinct phenotypic and transcriptional signatures induced by pregnancy in N+P vs. S+P T_FGS_ also prompted us to investigate whether this resulted in differential susceptibility to NFAT inhibition, which has been reported to hinder the induction of T cell exhaustion signatures^17^. Using FK506 as a pharmacological inhibitor of NFAT, we showed that FK506 treatment hindered the expression of exhaustion markers in N+P T_FGS_, whereas S+P T_FGS_ maintained an exhaustion phenotype despite FK506 treatment (Fig. 6b, Extended Data Fig. 6). These observations further support that the induction and maintenance of T cell exhaustion signatures by pregnancy may occur through distinct mechanisms in naive vs. memory T_FGS_.

### Pregnancy programs extensive chromatin remodeling in memory T_FGS_, but not naive T_FGS_

**Extended Data Fig. 7:**
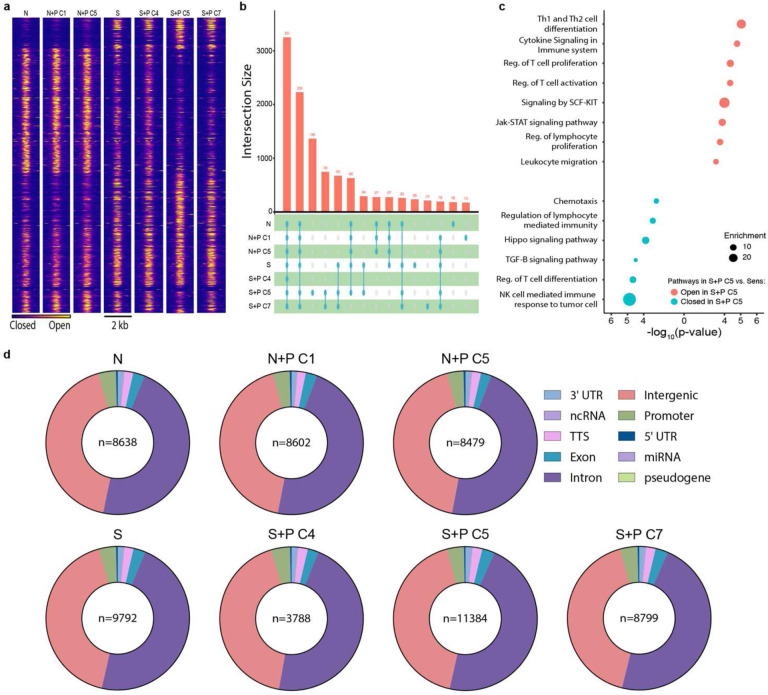
Peak distribution and visualization for T_FGS_ ATAC-Seq dataset. **a**, Chromatin accessibility heatmaps to further visualize global differences between T_FGS_ subsets. **b,** Upset plot showing the total number of reproducible peaks shared by various combinations of T _FGS_ subsets. This graph serves the same purpose as a Venn diagram, but maintains visual proportionality even when comparing across a large number of groups. **c**, Metascape pathway/gene ontology analysis for differentially accessible peaks in S+P C5 vs. Sensitized T_FGS_. **d**, Pie charts showing the genomic distribution of reproducible ATAC-Seq peaks identified for each T_FGS_ subset.

It is well-established that CD8^+^ T cells undergo epigenetic modifications during both conventional effector responses and exhausted/dysfunctional responses that promote the stability of effector and dysfunctional phenotypes^25–29^. Therefore, we tested the hypothesis that pregnancy epigenetically reprograms naive and memory T_FGS_ to maintain distinct states of exhaustion and hypofunction. To test this, we used the same sorting strategy as described above for RNA-Seq to perform the Assay for Transposase-Accessible Chromatin with high-throughput sequencing (ATAC-Seq) and assess the chromatin accessibility profiles of T_FGS_. As demonstrated by UMAP, Sensitized vs. Naive T_FGS_ possessed distinct chromatin states, and the chromatin states of N+P C1 and S+P C4 subsets resembled Naive and Sensitized T_FGS_, respectively (Fig. 7a, Extended Data Fig. 7–b). The chromatin state of N+P C5 was surprisingly similar to that of naive T_FGS_, suggesting that pregnancy induced minimal epigenetic modifications in naive T_FGS_. A limited set of peaks opening and closing was observed between S+P C5 vs. Sensitized T_FGS_ (n=417 opening and n=496 closing) (Fig. 7b, Extended Data Fig. 7–b).

**Extended Data Fig. 8:**
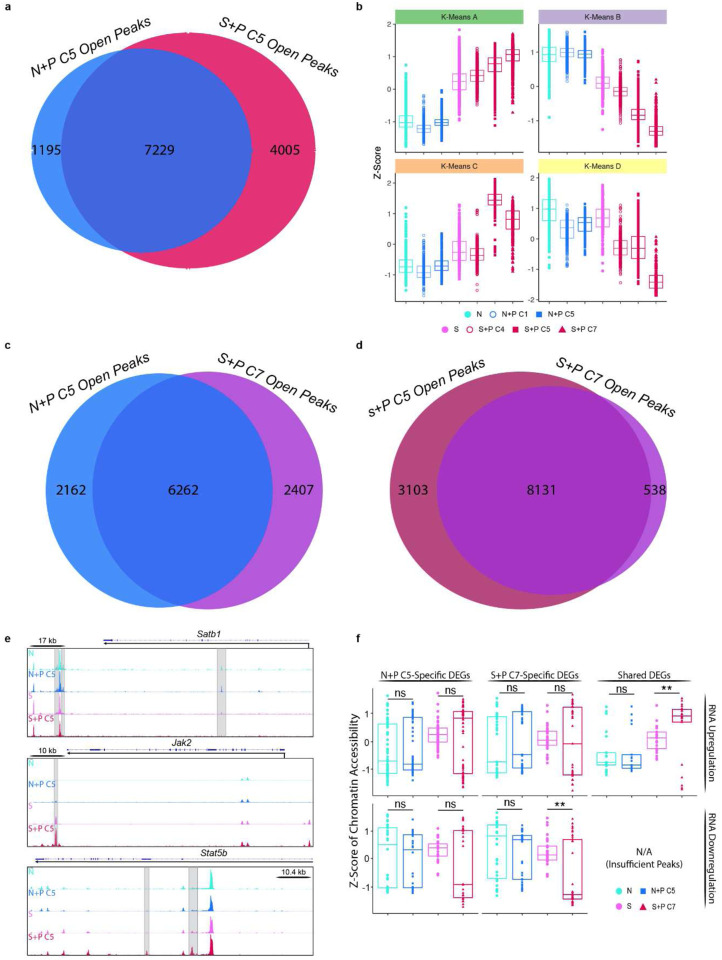
Comparison of pregnancy-induced chromatin remodeling in naive vs. memory T_FGS_. **a,** Venn diagram directly comparing the chromatin accessibility of N+P C5 vs. S+P C5. **b**, Box plots visualizing relative expression of DAPs in each K-Means cluster (K-Means A-D) identified in Fig. 5A. **c-d**, Venn diagram directly comparing the chromatin accessibility of N+P C5 vs S+P C7 (**c**), and S+P C5 and S+P C7 (**d**). **e**, Additional ATAC-Seq tracks of the Satb1 (top), Jak2 (middle), and Stat5b (bottom) loci. Peaks uniquely induced or lost in S+P C5 highlighted in gray. **f**, Box plot of chromatin accessibility (C.A.) differences at the shared pregnancy-induced DEGs (identified in Fig 3A) between N+P C5 and S+P C7.

Remarkably, S+P C5 T_FGS_ exhibited extensive chromatin remodeling compared to N+P C5 T_FGS_ (Fig. 7–b, Extended Data Fig. 7–b). These differences were apparent when visualizing the Differentially Accessible Peaks (DAPs) via heatmap, where there was a large set of peaks opening and closing in S+P C5 vs. N+P C5 T_FGS_ (n=1390 opening and n=943 closing). To classify the epigenetic modifications induced by pregnancy in memory T_FGS_, we performed pathway analysis of the genes associated with DAPs in S+P C5 vs. Sensitized T_FGS_. This analysis revealed that pathways enriched in S+P C5 were associated with T cell signaling and differentiation, including Jak-STAT, SCF-KIT, and Hippo signaling pathways (Extended Data Fig. 7c). Extensive chromatin remodeling was also observed for S+P C7 (Extended Data Fig. 8–d).

We next addressed the potential confounder that the epigenetic modifications seen in S+P C5 but not N+P C5 actually occurred during skin sensitization rather than pregnancy, and that the chromatin accessibility profile of S+P C5 T_FGS_ may be more indicative of memory than exhaustion. Due to the level of variability within Sensitized samples in our ATAC-Seq dataset, a simple pairwise comparison of these two groups was not sufficient to rule out this possibility. Therefore, we leveraged insights obtained from our RNA-Seq dataset to assess chromatin remodeling associated with pregnancy-induced DEGs unique to either N+P C5 or S+P C5 T_FGS_ (Fig. 7–d). Notably, both of these gene sets were enriched for T cell exhaustion (Fig. 4). At the loci of DEGs (n=831) induced uniquely in naive T_FGS_ by pregnancy, we observed no significant change in chromatin accessibility between N+P C5 vs. naive, or S+P C5 vs. Sensitized T_FGS_ (Fig. 7c). Furthermore, at the loci of DEGs (n=817) induced in memory T_FGS_ by pregnancy, we again detected no significant changes in chromatin accessibility in N+P C5 vs. Naive T_FGS_. Importantly, significant increases and decreases in chromatin accessibility were observed in S+P C5 vs. Sensitized T_FGS_ at these loci, corresponding to transcriptional up- and down-regulation (Fig. 7d). Similar chromatin remodeling was also observed for S+P C7 T_FGS_ (Extended Data Fig. 8f). These observations suggest that the chromatin accessibility profiles of S+P-C5 and C7 are indeed associated with pregnancy, thus raising the possibility that pregnancy-mediated chromatin remodeling is a mechanism unique to memory T_FGS_, while the induction of exhaustion in naive T_FGS_ does not require chromatin remodeling.

We next used HOMER *de-novo* motif analysis to search for enrichment of conserved transcription factor DNA binding motifs associated with T cell function and differentiation among DAPs in S+P C5 vs. Sensitized T_FGS_. This analysis identified key motifs associated with master regulators influencing both early- and late-stage T cell exhaustion, including Nfatc1, Nfatc2, Tbx21, Eomes, Runx, and Jun (opening), and Nfatc2, Batf, Jun, Fos, and AP-1 (closing) (Fig. 7–f)^30^. Additionally, motifs enriched in closed peaks of S+P C5 also included important hallmarks of T cell memory Lef1, Tcf7, and Tcfl2 (Fig. 7f). The presence of exhaustion-associated motifs in both open and closed peaks of S+P C5 T_FGS_ suggests that these cells are in an intermediate state of exhaustion rather than terminally exhausted, an interpretation consistent with the partial reduction in cytokine production observed in Fig. 6a. Furthermore, these data suggest that the extensive epigenetic reprogramming of memory T_FGS_ during pregnancy may encompass not only the accession of genes involved in the maintenance of T cell exhaustion, but also critical reversal of the conventional memory T cell signature.

**Extended Data Fig. 9:**
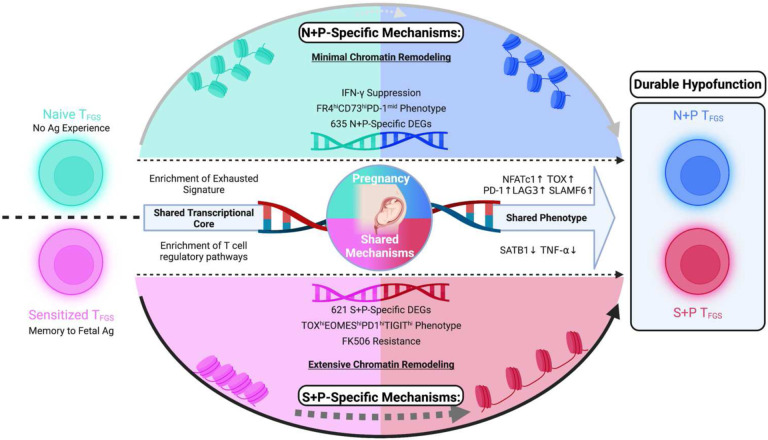
Pregnancy adaptively utilizes multiple mechanisms to induce dysfunction in memory and naive T_FGS_. Graphical abstract. Pregnancy induces a shared set of modifications in both naive and memory T_FGS_, including transcriptional exhaustion, reduced cytokine production, and T cell hypofunction. However, substantial phenotypic and transcriptional differences were observed between N+P and S+P T_FGS_, indicating that reprogramming of naive vs. memory T_FGS_ during pregnancy is mechanistically distinct. Finally, pregnancy mediates profound chromatin remodeling to induce hypofunction in memory T_FGS_, but these epigenetic modifications are not observed in naive T cells after pregnancy.

**Figure 8:**
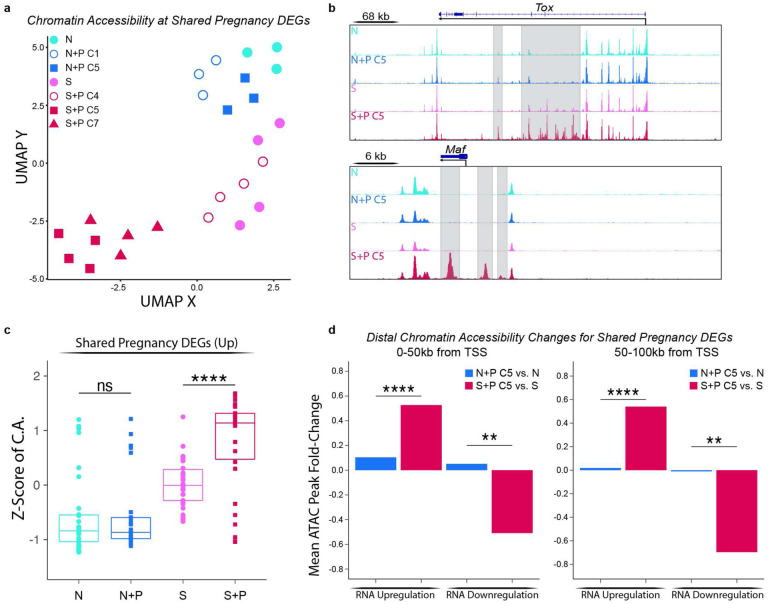
Extensive chromatin remodeling occurs at shared exhaustion-associated loci in post-partum memory T_FGS_, but not naive TFGS. **a**, UMAP plot comparing chromatin accessibility at the 196 DEGs shared by both N+P C5 and S+P C5. **b**, ATAC-Seq tracks at the Tox (top) and Maf (bottom) loci. Peaks uniquely induced in S+P C5 are highlighted in gray. **c**, Box plot of chromatin accessibility (C.A.) differences at the 196 shared pregnancy-induced DEGs. **d**, Bar plots visualizing the mean fold-change of distal ATAC-seq peaks within 0–50kb (left) or 50–100kb (right) of the TSS of shared pregnancy-induced DEGs. Fold-changes of peak accessibility are shown as N+P C5 vs. Naive (blue) or S+P C5 vs. Sensitized (red). Data acquired from 2 or more biologically independent experiments. P values were determined by Welch’s t-test. ns: not significant; **P<0.01; ****P<0.0001.

Finally, to confirm that pregnancy-mediated epigenetic reprogramming occurs uniquely in memory T_FGS_ and is not detectable in naive T_FGS_, we focused our analysis on the 196 shared DEGs induced by pregnancy in both N+P C5 and S+P C5 T_FGS_ (Fig. 3). Visualizing the chromatin accessibility of these loci via UMAP and box plots clearly demonstrated the significant epigenetic remodeling present in S+P C5 vs. Sensitized T_FGS_, but not in N+P C5 vs. Naive T_FGS_ (Fig. 8a+c). These differences are also readily apparent when visualizing individual exhaustion-associated loci such as *Tox* and *Maf,* where multiple open peaks are present in S+P C5 T_FGS_ but not in Sensitized or N+P C5 T_FGS_ (Fig. 7b, Extended Data Fig. 8e). Analyzing the chromatin accessibility of shared DEGs between S+P C7 and N+P C5 produces similar results (Extended Data Fig. 8f). Notably, this pregnancy-mediated chromatin remodeling remained detectable at distances of up to 100kb from the transcription start sites of these loci, supporting the possibility of both proximal remodeling of the locus itself along with distal enhancer remodeling (Fig. 7d). Taken together, we conclude that pregnancy induces significant chromatin remodeling to reprogram memory CD8^+^ T cells towards transcriptional and phenotypic exhaustion and increased susceptibility to transplantation tolerance induction, while naive CD8^+^ T cells undergo minimal epigenetic modification to achieve this hypofunctional state (Extended Data Fig. 9).

## Discussion

Pregnancy is an immunological paradox, where the conflict between robust immunity towards foreign pathogens and tolerance to the semi-allogeneic fetus must be simultaneously resolved in order to preserve the survival of the species. The imperative to preserve fetal viability underscores the necessity of multiple redundant mechanisms to achieve fetal tolerance. In this study, we demonstrate the remarkable ability of pregnancy to restrain not only naive, but also memory T cell responses towards the semiallogeneic fetus to achieve full-term delivery of viable offspring, even after sensitization via prior rejection of a fully mismatched paternal allograft. We identified a core transcriptional signature of 196 DEGs induced by pregnancy in both naive and memory T_FGS_ which was corroborated with phenotypic evidence, and showed that this signature was highly enriched for CD8^+^ T cell exhaustion.

We revealed that the majority of the phenotypic and transcriptional signatures induced in naive and memory T_FGS_ following encounter with the semi-allogeneic fetus were unique. As a result, the overall state of hypofunction achieved differed between naive and memory T_FGS_. Whereas post-partum naive T_FGS_ displayed a complete inhibition of proinflammatory cytokine production, post-partum memory T_FGS_ were only partially inhibited for TNF-α, and not for IFN-γ. Conversely, post-partum memory T_FGS_ were more robustly protected than naive T_FGS_ against the loss of exhaustion markers in the presence of NFAT inhibition (FK506). Importantly, pregnancy relieved the barrier memory T_FGS_ normally pose to transplantation tolerance, as evidenced by the enhanced survival of subsequent offspring-matched heart grafts in S+P recipients under co-stimulation blockade. The potential mechanistic significance of these nuanced differences between post-partum memory vs. naive T_FGS_ may be explained by the observation that pregnancy induced profound chromatin remodeling at exhaustion-associated loci exclusively in memory CD8^+^ T_FGS_, whereas naive T_FGS_ remained epigenetically unmodified during pregnancy. This difference in epigenetic signature may provide an explanation for the enhanced resistance to FK506 treatment seen in S+P T_FGS_. We speculate that transcriptional and epigenetic differences could be explained by differential responses to identical pregnancy-induced cues in naive vs. memory CD8^+^ T cells, differences in migratory behavior between naive and memory CD8^+^ T cells that dictate divergent access to pregnancy-induced cues, or by a combination of both factors.

Stable epigenetic programming has been reported in CD8^+^ T cells during the establishment of both memory and exhaustion^25–29^. We report that pregnancy-induced increases in chromatin accessibility in S+P T_FGS_ were enriched for transcription factor motifs implicated in both early- and late-stage T cell exhaustion, whereas motifs enriched in reduced chromatin accessibility included important hallmarks of T cell memory. These data raise the tantalizing possibility that pregnancy utilizes targeted epigenetic manipulation in memory T_FGS_ not only to induce transcriptional exhaustion, but also to silence pre-existing memory/effector programming. Taken together, our studies reveal a novel endogenous mechanism for the reprogramming of antigen-specific memory T cells towards exhaustion and hypofunction. This insight is of critical relevance for the success of transplantation tolerance in the clinic, where the conceptual framework for reprogramming of memory donor-specific T cells heretofore has not been identified^9–11^.

Finally, viewing CD8^+^ T cell exhaustion/hypofunction through the lens of pregnancy potentially solves the seemingly counterintuitive evolutionary puzzle of why exhaustion is so quickly induced when T cells are exposed to chronic infections or tumors, which is often detrimental to the host. We theorize that this timeline is imposed by mammalian pregnancy requiring a rapid restraint of fetus-specific alloreactive T cells to preserve fetal viability. Moreover, while the phenotype and transcriptome of exhaustion was first discovered in the context of chronic infection and tumors, we posit that this phenomenon should be reevaluated from the perspective that exhaustion pathways developed under the stringent need to preserve the semi-allogeneic fetus, and these mechanisms were subsequently hijacked by chronic infections and tumors. Thus, insights into how exhaustion is programmed into naive and memory T_FGS_ during pregnancy are relevant not only to addressing problems related to high-risk pregnancies and transplantation tolerance, but also to broader clinical issues such as autoimmunity, chronic infection, and cancer, where controlling T cell hypofunction is also desirable.

## Methods

### Mice.

Eight- to twelve-week-old female C57Bl/6 (B6, H-2^b^) mice were purchased from Harlan Laboratories. *Act-2W-OVA* transgenic mice on a B/6 background (2W-OVA.C57BL/6) were a gift from James Moon (Massachusetts General Hospital, Harvard Medical School, Charlestown, Massachusetts, USA). Donor/paternal 2W-OVA.BALB/c (2W-OVA.B/c, H-2^d^) mice were backcrossed from 2W-OVA.B/6 mice for >10 generations. For semi-allogeneic pregnancies, a harem was formed in which a male 2W-OVA.BALB/c was introduced to 4 virgin B/6 females. Approximately 50% of F1 from this mating were confirmed to be 2W-OVA-positive, and 2W-OVA.F1 (B6×2W-OVA.B/c) mice were used as heart donors. All animal experiments were approved by the Institutional Animal Care and Use Committee at the University of Chicago and adhered to the standards of the NIH Guide for the Care and Use of Laboratory Animals.

### Adoptive transfer, heart and skin transplantation.

For adoptive transfer experiments, ~4–12×10^**6**^ CD45.2^**+**^ CD8^**+**^ T cells, isolated via magnetic enrichment, were transferred retro-orbitally (r.o.) into naive CD45.1^**+**^ C57BL/6 hosts one day prior to heart transplantation. See below for T cell enrichment description. Heterotopic heart transplantations were performed as previously described^31^, by grafting 2W-OVA.F1 (B6×2W-OVA.B/c) hearts onto the inferior vena cava and aorta of female recipients. Tolerance (CoB/DST) was induced with a combination of anti-CD154 (MR1, BioXCell) at a dose of 500μg on day 0 (i.v.), and 250μg on days 7 and 14 (i.p.) post-transplantation, in combination with 2×10^**7**^ donor spleen cells on day 0. Graft survival was assessed by palpation 2–3 times per week, and the day of rejection was defined as the last day of detectable heartbeat. Flank skin from 2W-OVA.BALB/c was transplanted onto the B/6 mice.

### FK506 injection.

FK506 was injected daily (1 mg/kg i.p.) into pregnant mice beginning 5 days after the first observation of a copulation plug and ending on the date of euthanasia (day 0–3 post-delivery).

### T Cell enrichment.

Single-cell suspensions from spleens and pooled LNs (brachial, inguinal, and axillary) of individual mice were prepared for each experiment (see below). For flow cytometry and cell sorting assays, Pan-T lymphocytes were enriched with Pan-T cell isolation kit II (Miltenyi Biotech). For CD8^+^ T cell adoptive transfer experiments, the CD8α^+^ T Cell isolation kit (Miltenyi Biotech) was used instead. Samples were passed through LS columns on a QuadroMACS separator (Miltenyi Biotech) in MACS buffer (2%FBS + 2mM EDTA)

### Cell harvest and fluorescent staining for flow cytometry and cell sorting.

Spleens and LNs were harvested and passed through a 40μm cell strainer (Corning Inc., USA), followed by lysis of red blood cells via 2-minute incubation with ammonium chloride-potassium (ACK) lysis buffer (Quality Biological). After magnetic enrichment for T cells (see above), approximately 2×10^7^ cells were stained with a fixable live/dead stain (Invitrogen), followed by tetramer staining. Tetramer staining was performed for 35–45 min at room temperature with PE- and APC-conjugated OVA (SIINFEKL):H-2K^b^ tetramers (NIH Tetramer Core Facility). The cells were then stained for extracellular antibodies for 15–20min at 4^o^C. Samples were fixed with the Invitrogen Fix/Perm buffer kit according to the manufacturer’s instruction. Finally, fixed and permeabilized samples were stained for intracellular markers overnight. For phenotypic analysis, samples were acquired via flow cytometry after fixation and intracellular staining. For cell sorting, samples were sorted into RPMI after extracellular staining.

### In vitro stimulation and staining for IFNγ and TNFα.

Splenocyte stimulators from 2W-OVA.F1 mice were prepared and their red blood cells were lysed with ACK lysing buffer (Quality biological), followed by 30min incubation with anti-CD90.2 (53–2.1, BD Biosciences) to deplete T cells. Labeled T cells were depleted with two consecutive 35min incubations with rabbit complement (Cedarlane) at 37°C. >40× 10^6^ T-depleted splenocytes were then incubated overnight with 20μg/ml LPS. The following day, 1 × 10^6^ responder cells (Pan-T enriched splenocytes) were plated with 0.5 × 10^6^ stimulators (T-depleted APC’s) in triplicate in a 96-well plate (Corning) and incubated at 37°C overnight. Next, Golgi Plug (BD Biosciences) was added at 1:1000 and incubated for an additional 6h at 37°C. Live/Dead and extracellular staining were performed for 10min and 15min (respectively) on ice, and cells were then fixed with BD Cytofix/Cytoperm according to the manufacturer’s instruction (BD Biosciences). Finally, cells were stained for intracellular IFNg and TNFa and acquired via flow cytometry.

### Flow cytometry and cell sorting acquisition and analysis.

Flow cytometry samples for phenotypic panels and in-vitro cytokine stimulation assays were acquired on a Cytek Aurora flow cytometer (5 lasers, 16UV-16V-14B-10YG-8R). For cell sorting, samples were acquired and sorted on a BD Aria II 4–15 (70μm nozzle), BD Aria Fusion 5–18 (70μm nozzle), or the Invitrogen Bigfoot (100μm nozzle). The associated software for each cytometer is as follows: Aurora is Cytek SpectroFlo, Aria and Aria Fusion are BD FACSDiva, and Bigfoot is Invitrogen Sasquatch Software (SQS). Data were analyzed and visualized with FlowJo software v10.8.1 (FlowJo, LLC).

### Fluorescent antibodies for flow cytometry and cell sorting.

Fluorochrome-conjugated antibodies were used to select and sort cell subsets, analyze T cell phenotypes, and to determine cytokine production. The following antibodies were used in this study, separated by manufacturer (clone is indicated in parentheses). *Biolegend:* Ki67-PacificBlue (16A8), CD62L-BV510 (MEL-14), CD73-BV605 (TY/11.8), CD44-FITC (IM7), PD1-PEDazzle594 (RMP1–30), TIGIT-PECy7 (1G9), LAG3-BV785 (C9B7W), IFNγ (XMG1.2), TNFα-PECy7 (MP6-XT22), SATB1-AlexaFluor594 (O96C6), TIM3-APC/Fire750 (B8.2C12), OX40-BV711 (OX-86), OX40L-PECy7 (RM134L), Tim3-PerCP/Cy5.5 (B8.2C12), CD8-FITC (53–6.7), CD90.2-PECy7 (30H12), CD90.2-PerCP/Cy5 (53–2.1), CD4-APCCy7 (RM4–5). || *BD Biosciences:* CD90.2-BUV395 (53–2.1), CD4-BUV496 (GK1.5), CD19-BUV661 (1D3), CD11c-BUV661 (N418), F4/80-BUV661 (T45–2342), NK1.1BUV661 (PK136), TER119-BUV661 (TER-119), CD127-BUV737 (SB/199), CD8-BUV805 (53–6.7), FR4-BV421 (12A5), CTLA4-APCR700 (UC10–4F10–11), NK1.1-eFluor450 (PK136), Ter-119-eFluor450 (Ter119), Rorγt-BV650 (Q31–378), CD62L-BV605 (MEL-14). || *Invitrogen:* FoxP3-AlexaFluor532 (FJK-16s), CD44-BUV737 (IM7), PD1-SB780 (J43), TOX-eFluor660 (TXRX10), EOMES-PerCP/eFluor710 (Dan11mag), F4/80-eFluor450 (BM8), CD49b-eFluor450 (DX5), CD11c-eFluor450 (N418), PD1-PerCPe710 (J43). || *Santa Cruz Biotechnologies:* NFATc1-AlexaFluor488 (7A6), CD30L-AlexaFluor680 (RM153).

### RNA-Sequencing Data Collection and Processing.

RNA-Seq libraries were generated and amplified according to the SmartSeq2 protocol^32^. 200 live cells per sample/subset were sorted into 96-well optical PCR plates (Thomas Scientific) containing 4μl of lysis buffer at 4°C. cDNA sequencing libraries were generated using Nextera XT DNA Library Prep Kit and Nextera XT Index Kit (Illumina). All libraries were sequenced in the same run on a NovaSeq 6000 in a 150 bp/150 bp paired-end configuration. An average of ~55 × 10^6^ paired reads was generated per sample.

### RNA-Sequencing Data and Processing.

Raw RNA-Seq reads were trimmed for adapter content and filtered for truncated reads using Cutadapt v3.4^33^. Paired-end reads were aligned using STAR v2.6.1b^34^ against the GRCm39 (mm39) reference genome and transcriptome annotations, and non-uniquely mapping reads were removed. Per-sample read counts for each gene were quantified sample using featureCounts v2.0.1^35^.

### ATAC sequencing.

Chromatin profiling was performed by ATAC-seq as described previously^36,37^. In brief, ~3,000–50,000 sorted cells were washed in cold PBS and lysed to isolate intact nuclei. Transposition was performed at 37°C for 30min with the Tagment DNA Enzyme and Buffer kit (Illumina). After purification of the transposed DNA with the MinElute PCR purification kit (Qiagen), material was amplified via PCR for 13–14 cycles with Nextera XT Index primers (Illumina). Final product was purified again via MinElute PCR purification kit (Qiagen). Libraries were sequenced in the same run on a NovaSeq 6000 in a 150 bp/150 bp paired-end configuration. An average of 75 × 10^6^ paired reads was generated per sample.

### ATAC-Sequencing Data and Processing.

Raw ATAC-Seq reads were trimmed for adapter content and filtered for truncated reads using Cutadapt v3.4^33^. Paired-end reads were aligned using Bowtie2 v2.2.9^38^ against the GRCm39 (mm39) reference genome. Non-uniquely mapping reads and PCR duplicates were filtered with Bamtools v2.5 and Picard v2.21.8, respectively^39,40^. Peaks corresponding to ATAC-Seq cut sites for each sample were called using Genrich v0.6.1 in ATAC-Seq mode (https://github.com/jsh58/Genrich). Finally, reproducibly identifiable peaks for each experimental group were identified via ChIP-R v1.2.0^41^.

### Processing of ATAC-Seq peak set for differential accessibility analysis.

Reproducibly identifiable peaks across all experimental groups were merged into a single reference peak set using Bedtools v2.27.1^42^. Multibamsummary v3.5.1 from the Deeptools suite^43^ was used to generate per-sample read counts at each peak from the reference peak set. The read counts data was then imported into R v4.1.0, and each peak was assigned to a single gene via nearest TSS using GenomicRanges v1.46.1, ChIPpeakAnno v3.28.1, and the Org.mm.eg.db v3.14.0 genomic annotation object^44–46^.

### Sequencing Data Analysis and Visualization.

After completing data preprocessing as described above, the DESeq v1.34.0 package was used to conduct differential expression/accessibility analysis on sequencing datasets^47^. For both RNA-Seq and ATAC-Seq, the threshold for determining differential expression/accessibility was FDR (p_adj_)<0.1 and absolute value of log_2_ fold-change >0.9. In addition to DESeq2, we used current versions of the following packages for analysis and visualization (with description of purpose in parentheses). Viridis and RColorBrewer (color scale creation). Gplots, ggplot2 and ggrepel (graphing data and generating heatmaps). Uwot and VennDiagram (UMAP and Venn graphs, respectively). Tidyverse suite (dataset manipulation).

### ATAC-Seq motif analysis and locus visualization.

Motif analysis was performed by identifying unique and common peak sets between two experimental groups (using the reproducible peaks for each group as described above). These peak sets were then analyzed via HOMER de-novo analysis^48^ to search for significantly enriched motifs associated with ATAC-Seq cut sites and annotate these motifs to possible transcription factor targets. Individual loci were visualized by generating bigwig files for each sample and importing them into IGV v2.12.3^49^. A single track for each experimental group was created by summing the read counts of two representative samples from each group.

### Gene Set Enrichment Analysis.

GSEA software (4.0.3) was downloaded from the Broad Institute (https://www.gsea-msigdb.org/gsea/index.jsp), and pre-ranked GSEA was performed on the selected gene sets in this study. Gene set files were downloaded from the Molecular Signatures Database or prepared manually as gene matrix expression files (.GMX), using DESeq2 on published RNA-Seq data. Ranked gene lists for our transcriptional data were generated from by arranging genes based on the Change Metric (fold change × −log_10_ p_adj_) from high to low. The change metric combines both significance and intensity of expression changes, while preserving the direction (up- or -downregulation) with positive or negative values.

### Pathway Analysis.

Two lists of DEGs (or differentially accessible peaks) were created for each pairwise comparison, one for upregulated/opened regions, and one for downregulated/closed regions. The ENSEMBL gene IDs of each list were then uploaded to Metascape Pathway analysis^50^ to calculate the enrichment and significance of functional gene pathways from Gene Ontology (GO), Kegg, Reactome, or WikiPathways databases (primarily GO).

### Computational resources.

All data preprocessing for both ATAC-Seq and RNA-Seq (adapter trimming, alignment, filtering, generation of read-counts, and peak-calling) was performed on the Midway2 highperformance compute cluster, which is maintained by the University of Chicago Research Computing Center.

### Statistical analysis.

Statistical significance analyses were performed using GraphPad Prism version 9.2.0. Sample size of >5 animals per experiment were chosen to ensure adequate power. Graft survival significance was assessed using a Kaplan-Meier/Mantel-Cox log rank test. *P* values <0.05 were considered to indicate a significant difference. To calculate differences between experimental animals, we used Kruskal-Wallis test (ANOVA) with Dunn’s post hoc test for pairwise multiple comparisons, one-way ANOVA with Tukey’s post-hoc test, or Welch’s unpaired *t* test (specific tests for each subfigure are indicated in the figure legends). Asterisks used to indicate significance correspond to the following: *p<0.05, **p<0.01, ***p<0.001, and ****p<0.0001.

## Extended Data

## Supplementary Material

1

## Figures and Tables

**Figure 6:**
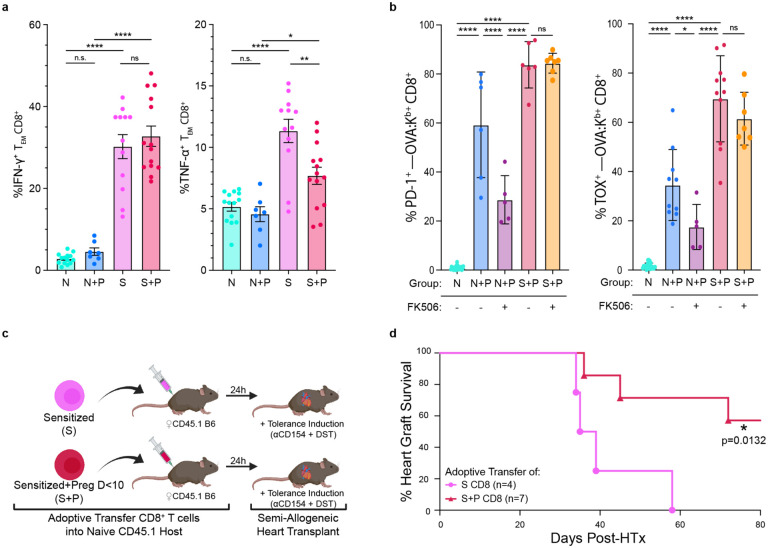
Pregnancy induces memory T_FGS_ to become hypofunctional. **a**, Bar graphs visualizing IFN-γ + (left) and TNF-α (right) production of CD 8 T_EM_ cells after overnight in-vitro stimulation with activated fetusmatched APCs. **b,** Expression levels of PD-1 (left) and TOX (right) in the presence or absence of FK506, an + inhibitor of NFAT. **c**, Experimental design for (**d**). CD8 T cells from Sensitized or S+P mice were adoptively transferred (AdTr) into naive CD45.1 B6 mice. 1 day after AdTr, these mice received allogeneic 2W-OVA.F1 (2W-OVA.B/c x B6) heart transplantation with anti-CD154+DST tolerance induction. **d**, Percentage of 2W-OVA.F1 heart graft survival among AdTr recipients; *n* = 4–7 per group. Flow cytometry data were acquired from 2 or more biologically independent experiments; *n* = 4–13 per group. Data represent mean ± SEM. P values were determined by Kruskal-Wallis test with Dunn’s post hoc test (**a**), Ordinary one-way ANOVA (**b**), and Mantel-Cox log-rank test (**d**); *P<0.05; **P<0.01; ****P<0.0001.

**Figure 6:**
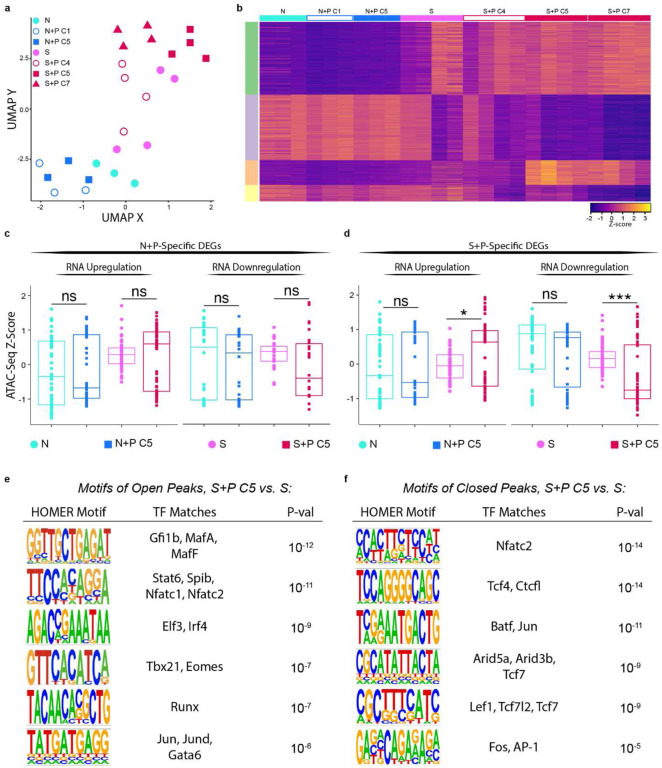
Pregnancy significantly alters the chromatin state of memory T_FGS_. **a-f,** T_FGS_ subsets were acquired and sorted for ATAC-Seq as in Fig. 2a; *n* = 3–4 per group. **a**, UMAP plot comparing global chromatin accessibility between sorted T _FGS_ subsets. **b**, Row-normalized ATAC-seq accessibility heatmap of the top differentially accessible peaks among T_FGS_ subsets, organized via K-means clustering (K-Means A-D). **c-d**, Box plots visualizing chromatin accessibility at DEGs unique to N+P C5 vs. N (**c**), or unique to S+P C5 vs. S (**d**) as identified in Fig. 3. **e-f**, HOMER de-novo analysis indicates nucleotide motifs associated with transcription factor binding that were significantly enriched in reproducible ATAC peaks opening (**e**) or closing (**f**) in S+P C5 vs. Sensitized T_FGS_. Data acquired from 2 or more biologically independent experiments. P values were determined by Welch’s t-test. ns: not significant; *P<0.05; **P<0.01; ***P<0.001; ****P<0.0001.

## Data Availability

The RNA-seq and ATAC-seq data have been deposited as a SuperSeries in the Gene Expression Omnibus (Accession code GSE216302). Additional information and materials will be made available upon request.
