# Supplementary material for: Pregnancy programs epigenetic and transcriptional exhaustion in memory CD8+ T cells
Source: Res Sq. 2023 Apr 5:rs.3.rs-2196637. Preprint. [Version 1] doi: 10.21203/rs.3.rs-2196637/v1 (PMC10104270; doi:10.21203/rs.3.rs-2196637/v1)
Supplement: 1 [file NIHPPRS2196637V1-supplement-1.pdf]

Panel 1			Panel 2	
**Fluorophore**	**Marker**		**Fluorophore**	**Marker**
BUV395	CD90.2		BUV395	CD90.2
BUV496	CD4		Live/Dead Blue	Live/Dead
BUV661	Dump		BUV496	CD4
BUV737	CD127		BUV661	Dump
BUV805	CD8		BUV737	CD44
BV421	FR4		BUV805	CD8
BV450 (PacBlue)	Ki67		BV421	FR4
BV510	CD62L		BV450 (PacBlue)	SLAMF6
BV605	CD73		BV510	CD62L
BV785	LAG3		BV605	CD73
FITC	CD44		BV650	RORyT
AlexaFluor 532	FOXP3		BV711	OX40
PerCP-Cy5.5	TIM3		SB780	PD-1
PE	OVA:Kb		AF488	NFATc1
PE-Dazzle	PD-1		AlexaFluor 532	FOXP3
Pe-Cy7	TIGIT		PerCP-e710	EOMES
APC	OVA:Kb		PE	OVA:Kb
APC-R700	CTLA4		AF594	SATB1
Live/Dead NIR	Live/Dead		Pe-Cy7	Ox40L
			APC	OVA:Kb
			eFluor660	TOX
			AF680	CD30L
			APC-Cy (Fire750)	TIM3

Supplementary Table 1: List of markers used in the two spectral flow cytometry panels used in this study.
